# Successful subtotal gastrectomy and hepatectomy for HER2-positive gastric cancer with liver metastasis after trastuzumab-based chemotherapy: a case report

**DOI:** 10.1186/s40792-024-01852-7

**Published:** 2024-03-05

**Authors:** Yuki Hirase, Takaaki Arigami, Yota Kawasaki, Daisuke Matsushita, Masataka Shimonosono, Yusuke Tsuruda, Ken Sasaki, Yoichi Yamasaki, Takahiko Hagihara, Hidetoshi Noma, Michiyo Higashi, Hiroshi Kurahara, Takao Ohtsuka

**Affiliations:** 1 Department of Digestive Surgery, Kagoshima University Graduate School of Medical and Dental Sciences, 8-35-1 Sakuragaoka, Kagoshima, 890-8520 Japan; 2 Department of Surgery, Terada Hospital, Kagoshima, Japan; 3 Department of Pathology, Kagoshima University Graduate School of Medical and Dental Sciences, 8-35-1 Sakuragaoka, Kagoshima, 890-8520 Japan

**Keywords:** Gastric cancer, HER2, Conversion surgery, Subtotal gastrectomy

## Abstract

**Background:**

Conversion surgery (CS) after chemotherapy is weakly recommended as a promising tool for improving prognoses in patients with unresectable gastric cancer. Moreover, several investigators have demonstrated the clinical efficacy of subtotal gastrectomy (sTG) with a small remnant stomach for the nutritional status and surgical outcome compared with total gastrectomy. Here, we report a patient with liver metastasis from human epidermal growth factor receptor 2 (HER2)-positive gastric cancer who underwent sTG and hepatectomy after trastuzumab-based chemotherapy.

**Case presentation:**

An 84-year-old male patient was diagnosed with HER2-positive gastric cancer with a single liver metastasis. He was treated with eight courses of trastuzumab in combination with S-1 and oxaliplatin as first-line chemotherapy. The primary tumor and liver metastasis shrank significantly. The metastatic liver lesion’s reduction rate was 65%. According to the Response Evaluation Criteria in Solid Tumors, the patient had a partial response. Therefore, he underwent an sTG with D2 lymphadenectomy and partial hepatectomy of segment 2. Histopathological examination revealed a grade 3 histological response without lymph node metastases from the primary tumor. No viable cancer cells were observed in the resected liver specimens. The patient received adjuvant chemotherapy with S-1. The postoperative quality of life (QOL) evaluated using the Postgastrectomy Syndrome Assessment Scale-45 was maintained, and the patient was still alive 8 months after the CS without recurrence.

**Conclusions:**

An sTG with a small remnant stomach might be clinically useful for preventing a decline in QOL and improving prognoses in patients with stage IV gastric cancer after chemotherapy.

## Background

Gastric cancer is the fourth most common malignancy and second leading cause of cancer-related deaths worldwide [1]. Currently, the Japanese Gastric Cancer Treatment Guidelines recommend systemic chemotherapy in patients with unresectable, advanced, or recurrent gastric cancer [2]. Remarkable advances in chemotherapy have led to high response rates [3]. In particular, the Trastuzumab (T-mab) for Gastric Cancer (ToGA) trial demonstrated the clinical efficacy of additional T-mab as a first-line regimen in patients with human epidermal growth factor receptor 2 (HER2)-positive unresectable advanced gastric cancer [4]. Consequently, trastuzumab-based chemotherapy is strongly recommended in patients with HER2-positive gastric cancer [2]. In contrast, an international retrospective cohort study showed that conversion surgery (CS) after chemotherapy for stage IV gastric cancer is safe and could be a new therapeutic tool to improve the survival of patients, especially those who underwent curative R0 resections [5]. Accordingly, CS after chemotherapy has been focused on as a promising surgical strategy in patients with stage IV gastric cancer [6–8].

Recently, the incidence of gastric cancer, which occurs in the upper third of the stomach, has increased in Asia [9]. Total gastrectomy (TG) is considered first for advanced tumors located in the upper third of the stomach. However, it is clinically difficult to maintain the quality of life (QOL) and nutritional status after TG in postoperative management [10]. Considering these issues, the clinical utility of a subtotal gastrectomy (sTG) with a small stomach remnant has been highlighted by several investigators [11–13]. Herein, we report a patient with HER2-positive gastric cancer and liver metastasis who underwent CS based on sTG and hepatectomy after trastuzumab-based chemotherapy.

## Case presentation

**Fig. 1:**
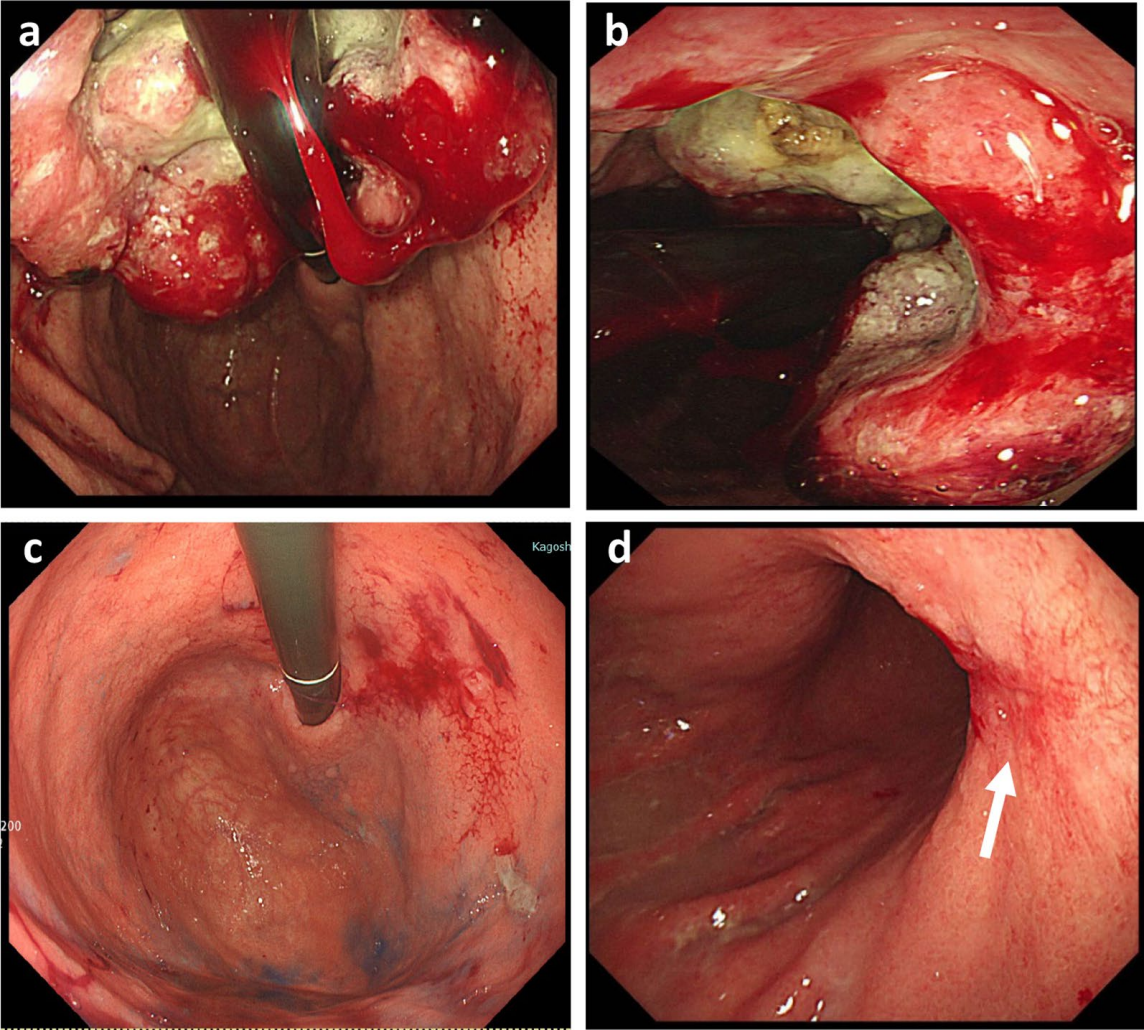
Esophagogastroduodenoscopy image. **a, b** Before chemotherapy. A type 2 tumor that extends from the middle to the upper third of the stomach is seen. **c, d** After eight courses of SOX plus T-mab. The tumor has disappeared and turned into a scar (arrow)

**Fig. 2:**
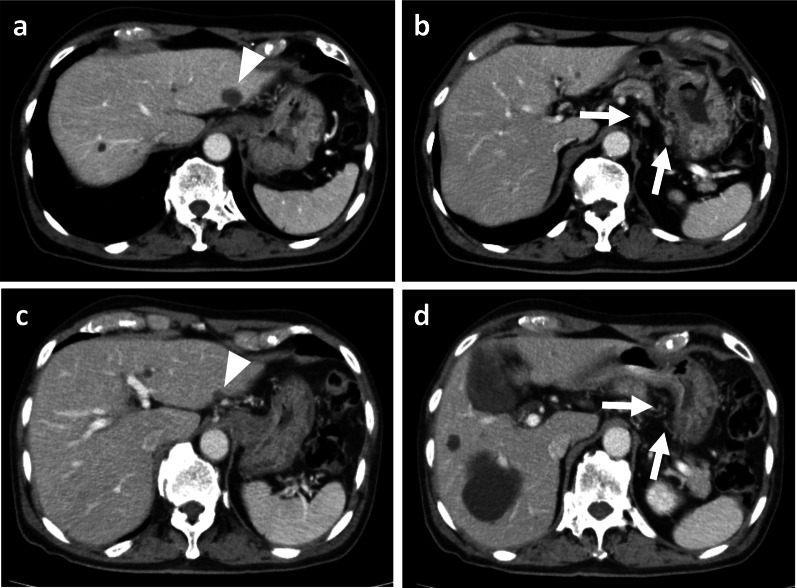
Enhanced computed tomography. **a, b** Before chemotherapy. Metastases at the station 3 lymph nodes (arrows) and segment 2 of the liver (arrowhead) are seen. **c, d** After eight courses of SOX plus T-mab. The lymph nodes (arrows) and liver metastasis (arrowhead) are remarkably reduced in size

**Fig. 3:**
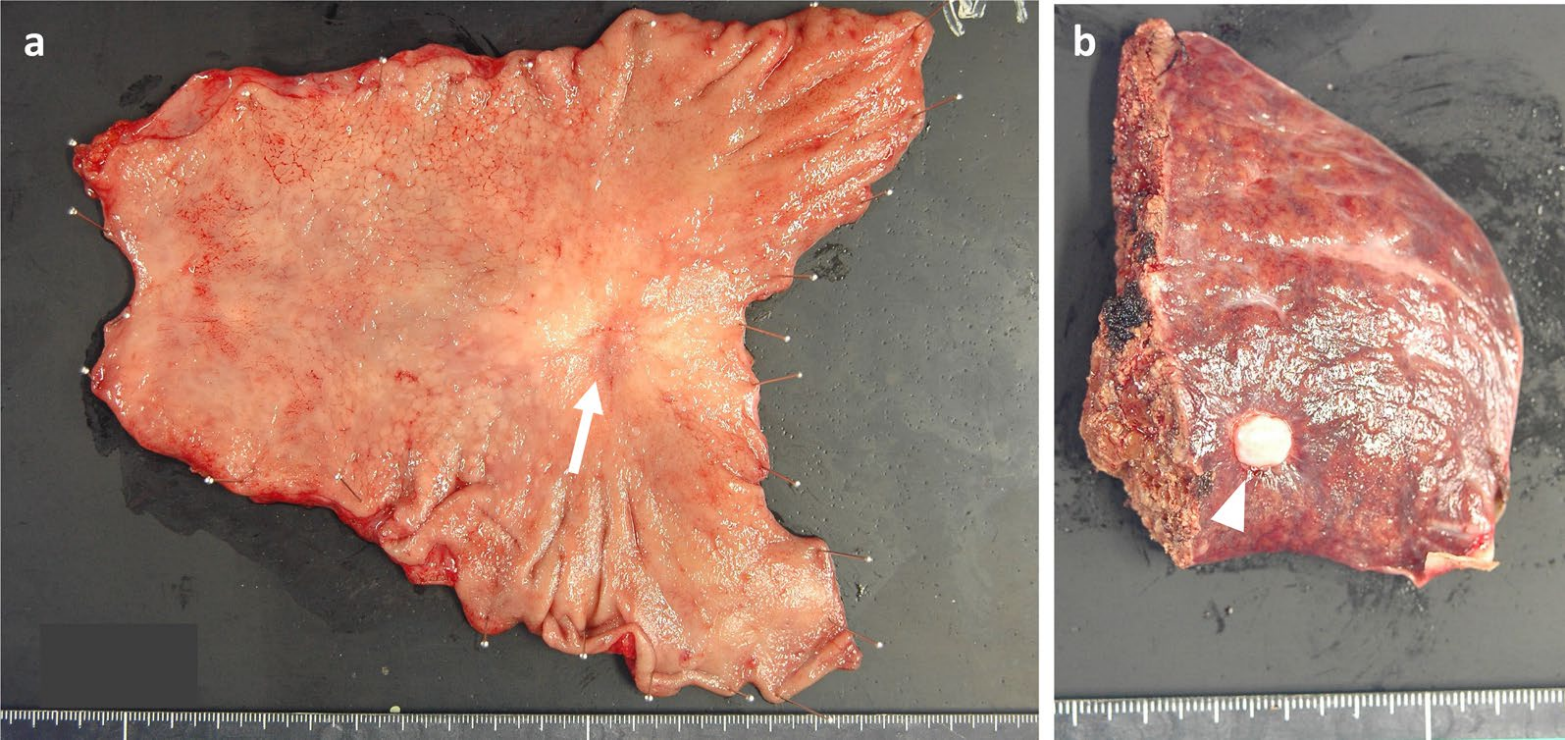
Macroscopic findings of the resected stomach (**a**) and liver (**b**). A scar of primary gastric tumor (arrow) and a white tumor at segment 2 of the liver (arrowhead) are identified

**Fig. 4:**
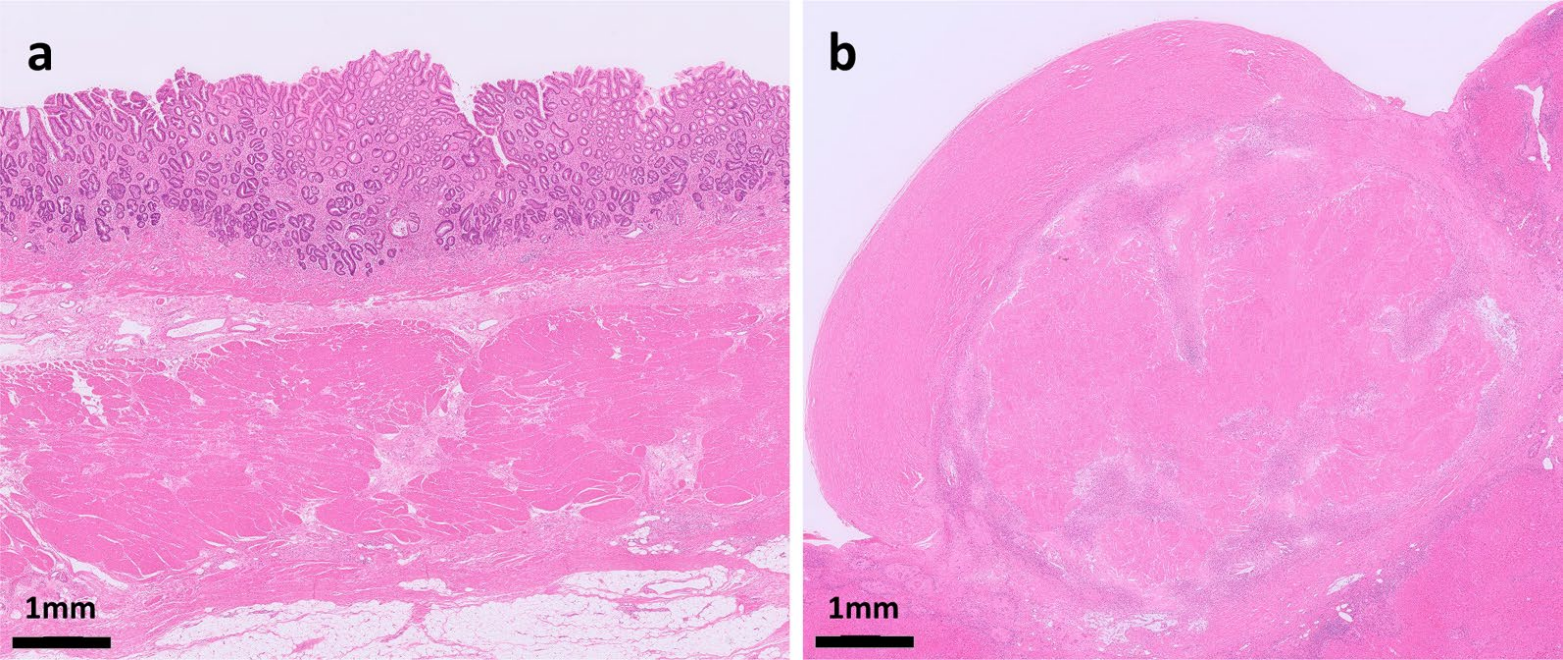
Histopathological findings. No viable tumor cells are seen in the specimens resected from stomach (**a**) and liver of segment 2 (**b**)

An 84-year-old male patient was diagnosed with gastric cancer on a screening esophagogastroduodenoscopy (EGD) performed at his local hospital. The EGD revealed a type 2 tumor extending from the middle to the upper third of the stomach (Fig. 1a, b). The histopathological examination of the biopsied specimens, including immunohistochemistry, revealed well-differentiated adenocarcinoma with an HER2 score of 3+. Enhanced computed tomography (CT) and magnetic resonance imaging (MRI) revealed enlarged lymph nodes at station 3 along the lesser curvature and a single liver tumor in segment 2 (S2) (Fig. 2a, b). Therefore, the patient was clinically diagnosed with stage IV HER2-positive gastric cancer (cT3N2M1). The patient received T-mab in combination with S-1 and oxaliplatin (SOX) as a first-line chemotherapy. This regimen consisted of a 3-week course of S-1 (80 mg/m^2^/day) orally on days 1–14, with oxaliplatin (130 mg/m^2^) and T-mab (8 mg/kg on the first course, followed by 6 mg/kg) intravenously on day 1. After eight courses of SOX plus T-mab, an EGD showed that the tumor had disappeared and had turned into a scar (Fig. 1c, d). Moreover, CT and MRI revealed shrinkage of the enlarged lymph nodes and liver tumor (Fig. 2c, d). The liver tumor’s reduction rate was 65%, indicating a partial response (PR), as determined by the Response Evaluation Criteria in Solid Tumors. Therefore, the patient was referred to our hospital for CS, considering the clinical indications. Body weight before CS was 56.9 kg. His serum levels of total protein and albumin before CS were 6.7 g/dL and 3.6 g/dL, respectively. As the distance from the esophagogastric junction to the tumor scar was approximately 4 cm, the patient underwent sTG with a small remnant stomach and D2 lymphadenectomy plus partial hepatectomy of S2. In the sTG, the transection line was selected to ensure a proximal margin of 3 cm from the tumor scar using intraoperative EGD. Furthermore, tumor-free involvement of the transection line was confirmed by intraoperative histopathological evaluation. The operative time was 364 min, with a blood loss of 359 ml. Macroscopically, a scar of the primary gastric tumor and a white liver tumor in S2 were identified (Fig. 3a, b). The histopathological examination revealed no residual tumors in the resected stomach or liver (Fig. 4a, b). Moreover, no tumor cells were observed in the dissected lymph nodes. These findings indicated a histopathologically complete response (CR), whereas the histological response of the primary tumor was classified as grade 3. The postoperative course was uneventful, and the patient was discharged on the eighth postoperative day. The patient received adjuvant chemotherapy with S-1, although the Postgastrectomy Syndrome Assessment Scale (PGSAS)-45 showed that the postoperative QOL was maintained [14]. Body weight and serum level of total protein were 58.0 kg and 7.4 g/dL at postoperative 6 months. The patient was alive 16 months after chemotherapy with no signs of disease recurrence 8 months postoperatively.

## Discussion

This report presents the case of an older adult patient with HER2-positive gastric cancer and liver metastasis who successfully underwent an sTG with a small remnant stomach and hepatectomy as CS after trastuzumab-based chemotherapy. To the best of our knowledge, this is the first case report focusing on the clinical utility of sTG as a surgical conversion strategy in older adult patients with stage IV gastric cancer.

HER2 is overexpressed in approximately 10–20% of patients with gastric cancer, and the presence or absence of HER2 positivity affects the recommended regimens in the clinical management of patients with unresectable advanced or recurrent gastric cancer [2, 15]. However, the ToGA trial showed that the overall tumor response rate and median survival time were 47% and 13.8 months, respectively, in patients with HER2-positive gastric cancer who received trastuzumab-containing chemotherapy [4]. The present patient had a PR, showing a 65% reduction in the liver tumor. Surprisingly, the histopathological examination revealed CR without tumor cells in the stomach, liver, or lymph nodes resected during CS. These results suggest the potential efficacy of T-mab as a molecular targeting agent in patients with HER2-positive gastric cancer.

The Japanese Gastric Cancer Treatment Guidelines weakly recommend CS for patients with stage IV gastric cancer who show an antitumor response after chemotherapy, patients with a preserved tumor response, and patients with R0 curative resections [2]. Arigami et al. retrospectively reviewed 44 patients with liver metastases from gastric cancer who received chemotherapy and reported that the 3-year survival rates among patients who underwent gastrectomies and hepatectomies, gastrectomies only, and nonsurgical treatments were 100%, 66.7%, and 0%, respectively [8]. Similarly, Oki et al. reported that the 3- and 5-year postoperative overall survival (OS) rates were 51.4% and 42.1%, respectively, in 94 patients with liver metastases who underwent CSs [6]. Furthermore, they suggested that hepatectomy was indicated for patients with a single liver metastasis and a nodal status of < N2 based on multivariate analysis for OS [6]. Because our patient had a single liver metastasis and a clinical nodal status of N0 after chemotherapy, he might have been a favorable candidate for CS. Although CS is a promising therapeutic option for improving prognosis, larger prospective studies are required to assess the prognostic significance of CS in patients with stage IV gastric cancer.

Although pathological examination revealed a histological response of grade 3, a high rate of disease recurrence is suspected due to malignant potential of stage IV before chemotherapy. The Japanese Gastric Cancer Treatment Guidelines recommend 1-year postoperative adjuvant chemotherapy with S-1 for pStage II gastric cancer [2]. Therefore, we are planning on 1-year administration of S-1 for the patient. According to the Japanese Gastric Cancer Treatment Guidelines, adjuvant chemotherapy is weakly recommended for stage IV gastric cancer with R0 resection [2]. However, there is no evidence based on clinical studies regarding drug regimens and the duration of adjuvant chemotherapy. Consequently, further prospective studies are needed to solve this issue.

Recent studies have demonstrated the clinical benefits of sTG with a small remnant stomach in patients with gastric cancer, including advanced-stage cancer [11–13]. In a retrospective study, Shimonosono et al. compared body weight changes, QOL, and prognosis between patients who underwent sTG (*n* = 26) and TG (*n* = 26) and showed that body weight loss after surgery was significantly greater in the TG group than in the sTG group (*P* < 0.01) [13]. Moreover, the PGSAS-45 showed that the sTG group had significantly improved QOLs compared to the TG group (*P* < 0.05) [13]. Three patients died of pneumonia in the TG group, whereas the OS was significantly longer in the sTG group than in the TG group (*P* = 0.01) [13].

Our patient was 84-year old and was at risk of pneumonia in the long-term after TG. Furthermore, even when sTG with a small remnant stomach was selected in this patient, we judged that curative R0 resection could be achieved. Therefore, the patient underwent an sTG with a small stomach remnant. The patient maintained a non-poor nutritional status and good QOL after CS. However, further studies with larger numbers of patients and longer follow-up periods are warranted to evaluate the clinical utility of sTG.

## Conclusions

Here, we present a case of stage IV HER2-positive gastric cancer in a patient who underwent an sTG and hepatectomy after trastuzumab-based chemotherapy, indicating a complete histopathological response. Our findings suggest that sTG with a small remnant stomach may be clinically useful in preventing a decline in QOL and improving prognosis.

## Data Availability

The datasets generated in this study are available from the corresponding author upon reasonable request.

## Abbreviations

CR: Complete response
CS: Conversion surgery
CT: Computed tomography
EGD: Esophagogastroduodenoscopy
HER2: Human epidermal growth factor receptor 2
MRI: Magnetic resonance imaging
PR: Partial response
QOL: Quality of life
S2: Segment 2
SOX: S-1 and oxaliplatin
sTG: Subtotal gastrectomy
TG: Total gastrectomy
T-mab: Trastuzumab
ToGA: Trastuzumab for gastric cancer
